# Promoter methylation of RASSF1A and DAPK and mutations of K-ras, p53, and EGFR in lung tumors from smokers and never-smokers

**DOI:** 10.1186/1471-2407-7-74

**Published:** 2007-05-03

**Authors:** Yang Liu, Weimin Gao, Jill M Siegfried, Joel L Weissfeld, James D Luketich, Phouthone Keohavong

**Affiliations:** 1 Department of Environmental and Occupational Health, University of Pittsburgh, Bridgeside Point, Suite 350, 100 Technology Drive, Pittsburgh, PA 15219, USA; 2 Department of Environmental Toxicology and The Institute of Environmental and Human Health, Texas Tech University, Lubbock, TX 79409, USA; 3 Department of Pharmacology, University of Pittsburgh, Pittsburgh, PA 15261, USA; 4 Department of Epidemiology, University of Pittsburgh, Pittsburgh, PA 15213, USA; 5 Department of Surgery, University of Pittsburgh, Pittsburgh, PA 15213, USA; 6 University of Pittsburgh Cancer Institute, Pittsburgh, PA 15213, USA

## Abstract

**Background:**

Epidemiological studies indicate that some characteristics of lung cancer among never-smokers significantly differ from those of smokers. Aberrant promoter methylation and mutations in some oncogenes and tumor suppressor genes are frequent in lung tumors from smokers but rare in those from never-smokers. In this study, we analyzed promoter methylation in the *ras-association domain isoform A (RASSF1A) *and the *death-associated protein kinase (DAPK) *genes in lung tumors from patients with primarily non-small cell lung cancer (NSCLC) from the Western Pennsylvania region. We compare the results with the smoking status of the patients and the mutation status of the K-*ras*, *p53*, and *EGFR *genes determined previously on these same lung tumors.

**Methods:**

Promoter methylation of the *RASSF1A *and *DAPK *genes was analyzed by using a modified two-stage methylation-specific PCR. Data on mutations of K-*ras*, *p53*, and *EGFR *were obtained from our previous studies.

**Results:**

The *RASSF1A *gene promoter methylation was found in tumors from 46.7% (57/122) of the patients and was not significantly different between smokers and never-smokers, but was associated significantly in multiple variable analysis with tumor histology (p = 0.031) and marginally with tumor stage (p = 0.063). The *DAPK *gene promoter methylation frequency in these tumors was 32.8% (40/122) and did not differ according to the patients' smoking status, tumor histology, or tumor stage. Multivariate analysis adjusted for age, gender, smoking status, tumor histology and stage showed that the frequency of promoter methylation of the *RASSF1A *or *DAPK *genes did not correlate with the frequency of mutations of the K*-ras, p53*, and *EGFR *gene.

**Conclusion:**

Our results showed that *RASSF1A *and *DAPK *genes' promoter methylation occurred frequently in lung tumors, although the prevalence of this alteration in these genes was not associated with the smoking status of the patients or the occurrence of mutations in the K-*ras*, *p53 *and *EGFR *genes, suggesting each of these events may represent independent event in non-small lung tumorigenesis.

## Background

Lung cancer, the leading cause of cancer-related death in the Unites States and worldwide, is strongly associated with tobacco smoke exposure [1]. In spite of extensive studies, the etiology of lung cancer remains poorly understood. Particularly, studies of lung cancer among never-smokers are few and little is known about the etiology of lung cancer in never-smoking patients. Some studies showed that the frequencies and/or patterns of genetic and epigenetic alterations in some genes in lung tumors from smokers differ from those in never-smokers. For instance, mutations in the K*-ras *oncogene, *p53 *tumor suppressor gene, and epidermal growth factor receptor (*EGFR*) gene have been found frequently in lung tumors and implicated in lung tumorigenesis. However, while K-*ras *mutations were detected primarily in lung tumors from smokers [2-4], the *EGFR *mutations were found mostly in lung tumors from never-smokers [5-7]. Furthermore, although the *p53 *mutations were detected in lung tumors from both smokers and never-smokers, the mutation types and spectra were different between the two groups of lung cancer patients [3,8].

In addition to mutations in oncogenes and tumor suppressor genes, results from previous studies demonstrated that promoter methylation of some genes occurred more frequently in lung tumors from smokers, compared with never-smokers [9-13]. Other genes whose transcription has been found frequently inactivated in lung tumors include the *ras-association domain isoform A (RASSF1A) *and the *death-associated protein kinase (DAPK) *genes, but the effects of tobacco smoking on the prevalence of promoter methylation of these genes in lung tumors remain unclear. The *RASSF1 *gene is located at 3p21.3 and functions as a tumor suppressor gene involved in cell apoptosis, genomic and microtubule stability, and cell cycle regulation [14]. This gene codes for two major transcripts, *RASSF1A *and *RASSF1C*, by alternative splicing [15,16]. Promoter methylation of the *RASSF1A *gene has been found in many human tumors, including those of the lung where it was detected in 30% to 50% of non-small cell lung cancer (NSCLC) [16,17]. The *DAPK *gene is located on chromosome 9p34.1. It encodes for a Ca+/calmodulin-regulated serine/threonine kinase involved in apoptosis induced by interferon-γ, tumor necrosis factor α, activated Fas, transforming growth factor β, ceramide and detachment of cells from the extracellular matrix [18]. *DAPK *suppresses tumor growth by increasing the occurrence of apoptosis [19]. Expression of *DAPK *is frequently lost in many human cancers, often as a result of silencing by DNA methylation. Promoter methylation of the *DAPK *gene has been found in 20% to 40% of NSCLC [20,21].

It has been suggested that epigenetic changes might addict cells to certain oncogenic pathways, predisposing cells to the accumulations of genetic mutations, which drives tumor progression [22]. On the other hand, overexpression of the K*-ras *gene can affect the activity of methytransferase [23,24] and potentially the methylation activity and regulation of gene transcription. Several studies have indicated the presence of an association between *RASSF1 *and Ras proteins. For instance, Ras binds RASSF1C in a GTP-dependent manner to mediate apoptosis [25]. Because *RASSF1A *has the identical Ras associate domain as *RASSF1C *[15,16], *RASSF1A *may bind Ras in the same manner as *RASSF1C*. Furthermore, Ras may also promote apoptosis through its association with a Nore-RASSF1-Mst 1 complex [26,27] since *RASSF1A *was found to be the closest Ras effector homologue to Nore1 in mammalian cells [15]. *RASSF1A *forms heterodimers with Nore1 through its non-homologous NH2-terminal segments and interacts with Ras [28]. Therefore, *RASSF1A *may serve as a Ras effector to mediate apoptosis.

DAPK and p53 may interact to mediate apoptosis. Raveh et al demonstrated that DAPK executes its anti-oncogenic action through activation of p53 in a mechanism that requires p19^ARF ^[29] and failure to perform apoptosis may result in tumorigenesis. Other studies showed that DAPK may also exert its p53-mediated suppressive effects on oncogenic transformation by imposing growth arrest, apoptosis or both [30,31].

Although *EGFR *mutations have been found frequently in lung tumors, the relationship between these mutations and promoter methylation in these tumors has not been well studied. *EGFR *is a 170 kDa trans-membrane glycoprotein. When *EGFR *binds to specific ligands, multiple signaling pathways are activated, including the RAS/RAF/ERK/MAPK pathway, resulting in cell proliferation, and the p13/AKT pathway [32]. Recently, it has been demonstrated that mutations in the tyrosine kinase domain region of the *EGFR *gene in NSCLC predict the response to tyrosine kinase inhibitors [33,34] and enable the identification of the subpopulation of lung cancers that are likely to respond to drugs targeted to *EGFR*s. The *EGFR *mutations were found consistently more common in never-smokers [5-7], indicating different pathways for lung tumorigenesis between smokers and never-smokers. Although both genetic and epigenetic alterations in oncogenes and tumor suppressor genes have been identified frequently in lung tumors, the interaction among these gene alterations and their significance in lung cancer remains poorly understood. In this study, we analyzed methylation in the promoter region of the *RASSF1A *and *DAPK *genes in 122 lung cancer patients. We compared the results with those reported by previous studies and with the status of mutations in the K*-ras*, *p53 *and *EGFR *genes in the same lung tumors obtained from patients from the Western Pennsylvania region.

## Methods

Lung tumor tissues analyzed in this study were obtained from 122 lung cancer patients, including 81 smokers and 41 never-smokers, all of whom were Caucasian and lived in the Western Pennsylvania region. All 122 tumors were among specimens collected between 1988 and 2001 and stored at the University of Pittsburgh Medical Center under an Institutional Review Board-approved protocol. The demographic and clinical profiles of these patients are shown in table 1. Of the 122 patients, 120 had NSCLC while 2 had SCLC, including one patient with carcinoid and one patient with neuroendocrine carcinoma. Most of the never-smokers were females (31/41, or 75.6%) while male patients accounted for 24.4% (10/41). The male and female patients among the smokers were 51.9% (42/81) and 48.1% (39/81), respectively.

Each lung tumor was histologically examined by a pathologist to identify the type and other clinical characteristics of the tumor. Any contamination of tumor cells by non-tumor cells was described by the pathologist. Most of the cells in these lung tumor samples were tumor cells. DNA was extracted from fresh-frozen tissues, using the Proteinase K treatment and phenol/chloroform method, and was dissolved in distilled water and stored at -20°C until use.

### Methylation-specific PCR (MSP)

Each genomic DNA sample was treated with sodium bisulfite (Sigma, Saint Louis, MO) and purified by using a Wizard DNA Clean-Up System (Promega Corporation, Madison, WI), as described previously [12]. Universal methylated human DNA (Chemicon International, Temecula, CA) was treated the same way and was used as a positive control DNA, and water was used as blank controls.

Two versions of MSP have been previously reported, including an original 1-stage MSP [35], and a modified 2-stage MSP that used 2 rounds of PCR and had a higher level of sensitivity to detect promoter methylation, compared with the original 1-stage MSP [36]. We first compared the sensitivity and validity of both MSP methods to analyze promoter methylation of these genes in DNA from some lung tumors. The results showed that promoter methylation was detected in the same tumors and at a similar frequency by using either the original or modified MSP for these genes, suggesting that the original 1-stage MSP was sufficiently sensitive to detect promoter methylation in lung tumors. However, in our experiments, the 2-stage MSP method showed MSP-products as one clear single band by gel electrophoresis analysis, while the 1-stage MSP method provided products that usually appeared with other minor bands (data not shown). Therefore, in this study, we applied a sensitive modified 2-stage MSP. The nucleotide sequences of the primers used for MSP analysis for both the *RASSF1A *and *DAPK *were the same as described by Belinsky et al [37]. During the stage-I PCR, the PCR amplification was carried out in a 25-μl reaction mixture containing 10 mM Tris-HCL (pH 8.3), 50 mM KCL, 2 mM MgCl_2_, 100 μM each dNTP, and 0.2 μM each primer. The reaction was heated at 95°C for 10 min., then amplified for 40 cycles [95°C/30 sec., 64°C (for *RASSF1A) *or 58°C (for *DAPK*)/30 sec., and 72°C/30 sec.], followed by a 10-min.-final extension at 72°C. Three microliters of each stage-I PCR product was diluted 10-fold with water and 1-μl was used for stage-II PCR, using the same reagents and conditions as for stage-I, except that the MgCl_2 _concentration was reduced to 1 mM and each sample was amplified in duplicated reaction, with one reaction containing primers specific for a methylated C and the other reaction containing primers specific for unmethylated C. Each reaction was heated at 95°C for 10 min., then amplified for 40 cycles each consisting, for the reaction containing methylated primers, of 95°C/30 sec., 68°C (for *RASSF1A*) or 66°C (for *DAPK*)/30 sec., and 72°C/30 sec., and, for the reaction containing unmethylated primers, of 95°C/30 sec., 64°C (for *RASSF1A*) or 68°C (for *DAPK*)/30 sec., and 72°C/30 sec., followed by a 10-min.-final extension at 72°C. Five microliters of each stage-II PCR product was separated on an 8% polyacrylamide gel. The gel was stained with ethidium bromide and photographed under UV illumination. The reproducibility of the results was confirmed by repeating the MSP analysis for each DNA sample. A portion of PCR products were purified using a MinElute PCR purification kit (QIAGEN, Valencia, CA) and directly sequenced to confirm methylation.

### Detection of K-*ras*, *p53*, and *EGFR *mutations

Data on mutations in K*-ras *(codons 12 and 13), *p53 *(exons 5 – 8), and *EGFR *(exons 18–21) genes were obtained from our previous studies [2,38,39].

### Statistical analysis

Chi-square test or Fisher's exact test was used in univariate analysis. Logistic regression models were used to assess the effect of multiple variables on methylation status and relationship between genetic and epigenetic alterations.

## Results

Overall, *RASSF1A *promoter methylation was detected in 46.7% (57/122) of the patients, a frequency that did not differ between smokers and never-smokers [48.1% (39/81) vs. 43.9% (18/41), p = 0.645], or between adenocarcinoma and the group of other tumor types [51.4% (37/72) vs. 40% (20/50), p = 0.210], or between stage-I and the group of other stages [51.9% (28/54) vs. 40.7% (22/54), p = 0.247]. Promoter methylation for *DAPK *was found in 32.8% (40/122) of the patients. When grouped according to the patient's smoking status, this gene methylation was detected in 34.6% (28/81) of the smokers and was not significantly different from the 29.3% (12/41) frequency observed among the never-smokers (p = 0.567). Furthermore, *DAPK *promoter methylation frequency did not differ between adenocarcinoma and the group of other tumor types [34.7% (25/72) vs. 30% (15/50), p = 0.583], or between stage-I and the group of other stages [31.5% (17/54) vs. 31.5% (17/54), p = 1.000].

Multivariate logistic regression models were employed to control for the potential confounding effects of variables, such as age, gender, smoking status, tumor histology and tumor stage on methylation status of *RASSF1A *and *DAPK *genes. As shown in Table 2, age, gender, and smoking status did not significantly affect the methylation frequency of the *RASSF1A *or *DAPK *gene. Promoter methylation of *RASSF1A *was associated significantly with adenocarcinoma [the group of other tumor types vs. adenocarcinoma: odds ratio (OR) = 0.661, 95% confidence interval (CI) = 0.454–0.962, p = 0.031], and marginally with early tumor stage (the group of other stages vs. stage-I: OR = 0.673, 95% CI = 0.444 – 1.022, p = 0.063). Neither tumor histology nor tumor stage was associated with *DAPK *promoter methylation.

Among the 122 patients, 32 (26.2%) had *p53 *mutations whereas 90 (73.8%) had wild-type *p53 *[38]. The frequency of *RASSF1A *promoter methylation in patients with *p53 *mutations was 56.3% (18/32) and was not significantly different from that in patients with wild-type *p53 *[43.3% (39/90), p = 0.212]. Likewise, the *DAPK *promoter methylation frequency in patients with *p53 *mutations was 25% (8/32) and not different from that in patients with wild-type *p53 *[35.6% (32/90), p = 0.331].

Thirty-six (29.5%) of the 122 patients had *K-ras *mutations and 86 (70.5%) had wild-type K*-ras *[2]. Among the K*-ras *mutation-positive patients, the frequency of *RASSF1A *promoter methylation was 41.7% (15/36) and was closely similar to the 48.8% (42/86) frequency in the group of patients with wild-type K*-ras *(p = 0.474). Likewise, the frequency of *DAPK *promoter methylation was closely similar between the K*-ras *mutation-positive patients and patients with a wild-type K*-ras *[30.6% (11/36) vs. 33.7% (29/86), p = 0.735].

Data for *EGFR *mutations were obtained from a previous study [39] and were available for only 70 of the 122 patients involved in this study, including 40 never-smokers and 30 smokers. *EGFR *mutations were detected in 14.3% (10/70) of the patients and exclusively among the never-smokers (10/40, or 25%), including 9 females and one male, and none among the smokers (p = 0.002), and inversely correlated with K-*ras *mutations [0% (0/10) vs. 28.3% (17/60), p = 0.049], in agreement with previous reports [6,7,13,40]. For comparison, *EGFR *mutations were found in both tumors with *p53 *mutations and those without these mutations [30% (3/10) vs. 28.3% (17/60), p = 0.912], suggesting that, unlike K*-ras *mutations, *p53 *mutations can occur in concert with *EGFR *mutations, in agreement with Kosaka's report (p = 0.4634) [6]. *EGFR *mutations did not correlate with promoter methylation of *RASSF1A *or *DAPK *(data not shown).

Of the 122 patients, 100 (81.9%) showed alterations of at least one gene, consisting of either promoter methylation of *RASSF1A *or *DAPK*, or mutations in the K-*ras *or *p53 *gene. Furthermore, since K*-ras *mutations were found mostly among smokers, smoking patients showed a higher fraction of tumors with gene alterations, compared with the never-smoking patients [87.6% (71/81) vs. 70.7% (29/41), p = 0.022]. Of the 100 patients with alterations of at least one gene, 46 (46%), 43 (43%), and 11 (11%) showed alterations affecting one gene, two genes, and three or more genes, respectively. The frequency of cases with more than one gene abnormality did not differ between adenocarcinoma and the group of other tumor types, or between stage I and the group of other stages (data not shown). The *EGFR *mutations were not included in the calculation because they were available for only 70 of the 122 patients.

## Discussion

The overall frequency of *RASSF1A *promoter methylation of 46.7% (57/122) in our study is similar to that found by Marsit et al [47% (83/178)] [17] and correlated significantly with adenocarcinoma (p = 0.031, table 2), in agreement with some previous studies [11,17,41], and marginally with tumor stage (p = 0.063, table 2). On the other hand, studies by Kim et al showed no such an association [42,43].

The overall frequency of *DAPK *promoter methylation of 32.8% (40/122) found in the present study is in line with that reported by Toyooka's 37% (14/38) [44] and was not associated with tumor histology (p = 0.111, table 2), in agreement with Kim's study [21], or with tumor stage (p = 0.348, table 2). Data from previous studies are conflicting. For instance, in Kim's study *DAPK *promoter methylation was associated with advanced stages NSCLC (p = 0.009) [21], whereas in Russo's study it was detected mostly in early stages NSCLC [45]. This discrepancy might be due to limited number of patients in Russo's study (n = 49) and/or tumor stage data that were not available for some patients in our study. The frequencies of both *RASSF1A *and *DAPK *gene promoter methylation were independent from the patients' smoking status (p = 0.990 for *RASSF1A *and p = 0.275 for *DAPK*, table 2), consistent with the results of previous studies [10,11,41,46].

There have been a few studies investigating the relationship between aberrant promoter methylation and mutations in oncogenes and tumor suppressor genes. Most of these studies compared promoter methylation of one or two genes with mutations in either the K-*ras *or *p53 *gene [41,43,46]. Only two recent studies investigated the relationship between mutations of multiple oncogenes and/or tumor suppressor genes and promoter methylation patterns but in lung cancer patients from Japan [13,40]. Here, we compared the promoter methylation status of *RASSF1A *and *DAPK *and mutations of K-*ras*, *p53 *and *EGFR *in the same lung tumors from lung cancer patients from the Western Pennsylvania region.

We observed no association of *RASSF1A *gene promoter methylation frequency with that of K*-ras *mutations (p = 0.474), in agreement with previous studies [11,41,43,46], or with the types of amino acid changes at codon 12 of the K*-ras *gene (data not shown). Activated Ras has been associated with enhanced proliferation, transformation, and cell survival through a series of effectors, including *RASSF1A*. However, because the *RASSF1A *gene is a tumor suppressor gene, it is likely that loss of *RASSF1A *expression by promoter methylation in NSCLC does not require *Ras *activation. Thus, our findings suggest that *RASSF1A *inactivation may induce malignant transformation by some distinct mechanisms other than Ras-mediated anti-apoptosis pathway, such as loss of genomic, microtubule stability and cell cycle regulation [14]. Furthermore, the prevalence of *DAPK *promoter methylation did not correlate with that of K*-ras *mutations (p = 0.735), consistent with previous data [11,21], suggesting that *DAPK *promoter methylation and K*-ras *mutations are independent events in non-small cell lung tumorigenesis.

The prevalence of *RASSF1A *promoter methylation in the present study did not correlate with that of *p53 *mutations (p = 0.212). Previously, Endoh et al found a similar trend in their study of 100 NSCLC patients (21/56 wild-type *p53 *vs. 21/44 mutant *p53*, p = 0.3165) [41]. On the other hand, Tomizawa et al observed a weak association between *RASSF1A *promoter methylation and *p53 *mutations (19/73 wild-type *p53 *vs. 16/37 mutant *p53*, p = 0.0842) [46]. The discrepancy may be in part due to the difference in tumor stages in the different studies, with all patients in stage I lung adenocarcinoma tumors in Tomizawa's study and a mixed of tumors of different types [adenocarcinoma: 67% (67/100) and 59% (72/122)] and stages [stage I: 57% (57/100) and 44% (54/122)] in Endoh's and our studies.

We observed no correlation between the prevalence of *DAPK *methylation and that of *p53 *mutations (p = 0.331), indicating *DAPK *promoter methylation and *p53 *mutations may be independent events in NSCLC. *DAPK*, and a few other genes, have been reported to operate upstream to *p53 *to induce apoptosis. For instance, Raveh et al found that the inactivation or loss of DAPK alone significantly reduces but not completely eliminates *p53 *in response to c-Myc or E2F-1 [29]. Kim et al found that *DAPK *promoter methylation and *p53 *mutations were not related in their study of 185 NSCLC patients (p = 0.85) [21]. Thus, our results are consistent with those of these studies [21,29], suggesting that *DAPK *may be wired simultaneously to a few independent upstream branches of *p53*, and may be involved in apoptosis without *p53 *participation.

Results of our study based on 70 patients showed that *EGFR *mutations did not correlate with the *RASSF1A *or *DAPK *gene promoter methylation. Previously, there are only two studies investigating the relationship of *RASSF1A *promoter methylation and *EGFR *mutation, reporting absent [13] or borderline [47] correlation between these two events. The relationship between these mutations and *DAPK *promoter methylation has not been previously investigated. Furthermore, there have been only two previous studies of epigenetic and genetic alterations in multiple genes in lung tumors, both involving lung cancer patients from Japan. In the study by Toyooka et al [13], mutations in the *EGFR *and K*-ras *genes were compared with promoter methylation of five genes in lung adenocarcinomas from 164 patients. There was no significant association between *RASSF1A *promoter methylation frequency and the K*-ras *or *EGFR *mutation frequency. Our data are in agreement with this finding. In the study by Suzuki et al [40], *EGFR*, *HER-2*, and K*-ras *mutations were compared with promoter methylation of nine genes in 150 NSCLC patients. The authors observed that *EGFR *mutation had an inverse correlation with promoter methylation of the secreted protein acidic and rich in cysteine and *p16 *genes. However, K*-ras *mutations were detected at a lower fraction of the tumor, compared with our study (6 out of 150 versus 36 out of 122) and was not investigated in relation to *RASSF1A *methylation.

The relationship between epigenetic and genetic alterations has also been investigated in other tumor types. For instance, in sporadic colorectal cancer and pancreatic cancer, *RASFF1A *promoter methylation was found predominantly in tumors with K*-ras *wild type [48,49]. Another study showed that sporadic colorectal cancer exhibiting microsatellite instability harbored concomitant *RASSF1A *gene promoter methylation and mutations in the K*-ras *and/or B-raf genes [50].

Taken together, results from these studies and our present study indicated the need for further investigation of the interaction between genetic and epigenetic alterations in lung tumors in an ethnically diverse patient population in order to further understand the development of lung cancer.

## Conclusion

Promoter methylation of the *RASSF1A *and *DAPK *genes was frequent in lung tumors from patients with primarily NSCLC, all of whom were Caucasian and from the Western Pennsylvania region. Furthermore, there was a significant association between promoter methylation of the *RASSF1A *gene and tumor histology, specifically adenocarcinoma. However, there was no significant association of the prevalence of promoter methylation of either gene with the smoking status of the patients or with the status of mutations in the K-*ras*, *p53*, or *EGFR *gene, suggesting each of these events may occur independently in lung tumorigenesis.

## Competing interests

The author(s) declare that they have no competing interests.

## Authors' contributions

YL carried out the methylation analysis, and drafted the manuscript; WG performed K-*ras *and *p53 *mutations and participated in the analysis and interpretation of data; JMS and JDL provided lung tumor samples, demographic/clinical information of patients and data interpretation; JLW performed statistical analysis and interpretation of data; PK designed and supervised the study, analyzed and interpreted data.

## Pre-publication history

The pre-publication history for this paper can be accessed here:



## Figures and Tables

**Figure 1:**
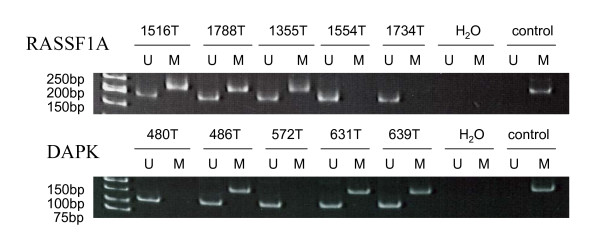
**Detection of *RASSF1A *and *DAPK *promoter methylation by MSP**. M indicates the presence of methylated *RASSF1A *or *DAPK*. U indicates the presence of unmethylated *RASSF1A *or *DAPK*. All samples were performed twice and representative data are shown.

**Table 1:** Demographic and clinical profile of 122 NSCLC patients

Variable	cases (%)
Age (mean ± s.d.*)	65.1 ± 10.2
Gender	
Male	52 (42.6)
Female	70 (57.4)
Smoking status	
Smoking	81 (66.4)
Never-smoking	41 (33.6)
Tumor types	
Adenocarcinoma	72 (59.0)
Squamous cell carcinoma	22 (18.0)
Bronchioloalveolar carcinoma	15 (12.3)
Adenosquamous carcinoma	9 (7.4)
Large cell carcinoma	2 (1.6)
Carcinoid	1 (0.8)
Neuroendocrine	1 (0.8)
Tumor stage	
I	54 (44.3)
II	24 (19.7)
III	19 (15.6)
IV	11 (9.0)
Unknown	14 (11.5)

*: standard deviation

**Table 2:** Logistic regression models of *DAPK *and *RASSF1A *promoter methylation (n = 122)

	DAPK		RASSF1A	
		
	OR (95% CI)	p	OR (95% CI)	p
Age	1.022 (0.977–1.068)	0.352	1.003 (0.964–1.045)	0.872
Sex	1.911 (0.746–4.894)	0.177	1.203 (0.513–2.822)	0.671
Smoking	1.743 (0.642–4.732)	0.275	1.006 (0.410–2.469)	0.990
Histology^†^	0.709 (0.465–1.082)	0.111	0.661 (0.454–0.962)	0.031
Stage^‡^	0.804 (0.509–1.269)	0.348	0.673 (0.444–1.022)	0.063

OR: odds ratio; CI: confidence interval.

†: the group of other tumor types vs. adenocarcinoma.

‡: the group of other stages vs. stage I.
